# Validation of self-reported diagnosis of diabetes in the 1946 British birth cohort

**DOI:** 10.1016/j.pcd.2014.05.003

**Published:** 2015-10

**Authors:** Silvia Pastorino, Marcus Richards, Rebecca Hardy, Jane Abington, Andrew Wills, Diana Kuh, Mary Pierce, the National Survey of Health and Development Scientific and Data Collection Teams

**Affiliations:** MRC Unit for Lifelong Health and Ageing at UCL, London, United Kingdom

**Keywords:** Diabetes, Validation, Questionnaire, Self-reported

## Abstract

The aim of this study was to validate self-reported diabetes and age at diagnosis among a sample of the British population, using general practitioners (GPs) as the reference standard. Using data from the Medical Research Council National Survey of Health and Development (NSHD), self-reported diabetes was determined either in response to a direct question at five follow-ups between 1977 and 2010, or from other self-reported medical information. A validation questionnaire was sent to the GP for all participants who reported a diagnosis of diabetes and gave permission to contact their GP (172). The validity of self-reported diabetes was assessed by calculating the percentage of self-reported diabetes cases that were confirmed by their GP, i.e. the positive predictive value (PPV). The difference between self-reported and GP-confirmed age at diagnosis was analysed with a Bland–Altman plot. Completed questionnaires were obtained from 157 GPs (91.2%). Of these, 149 confirmed their patient self-reported diabetes diagnosis (PPV = 94.9%). Results were similar when self-reported diabetes was assessed by responses to direct questions only (PPV = 95.4%). The average difference between self-reported and GP-reported age at diagnosis was 0.6 years (95% CI 0.2–1.1). We conclude that among the British population questionnaires are a valid method to assess GP-diagnosed diabetes, as measured by responses to a direct question or by patient-reported medical information.

## Highlights

• We used general practitioners (GP) to validate self-reported diabetes in the UK.
• 94.9% of 157 GPs confirmed their patient diabetes diagnosis.
• Mean difference between self-reported and confirmed age at diagnosis was 0.6 years.

## 1 Introduction

Several studies have assessed the validity of self-reported diabetes using either medical records or physical examination as the reference standard [1–13]. The agreement between self-reports and medical records, including family practitioners records [6,9,10,14], is usually good for conditions with well-defined diagnostic criteria, such as diabetes [9,10]. However, most validation studies of self-reported diabetes were based on a small number (<50) of diabetes cases [4,6,8] or were restricted to specific groups [1,7,8,11,13]. Furthermore none of these studies were conducted in the UK population. General practitioners (GP) are an optimal source of information on disease status in the UK as nearly all British citizens are registered with a GP practice. Moreover, during the last decade diabetes care in the UK has moved into general practice. The aim of this study was to evaluate the accuracy of participant-reported diabetes, and age at diagnosis, by comparing self-reported diabetes cases with GP-confirmed cases in a representative sample of the British population.

## 2 Subjects and methods

Data were taken from the Medical Research Council National Survey of Health and Development (NSHD). Details of the study have been described previously [14]. In brief, the NSHD is a socially stratified nationally representative sample originally consisting of 5362 single births in the first week of March 1946 in England, Scotland and Wales [14]. The cohort has been followed up 23 times between birth and the latest data collection at age 60–64 years [15]. The present study was based on information on hospitalisation from birth up to 60–64 years and of health questionnaires from the 1977, 1982, 1989, 1999 and 2006–2010 data collections. At the most recent follow-up, 3164 participants were still available for follow-up. Of these, 2661 (84%) provided information. Self-reported diabetes was determined in two ways. Firstly, in response to a direct question (at age 36 study members were asked: “Do you have diabetes all or most of the time?” at age 43, 53 and 60–64: “In the last ten years have you had diabetes? Has a doctor said you had this problem?”). Secondly, from all relevant medical information that study members reported. From birth, all hospital attendances and reasons for attending were recorded. Dates of diagnosis and medications were reported at 31, 36, 43 and 53 years.

Table 1 shows the follow-up process for the validation of self-reported questionnaires and the overall GP response rate. Of 230 study members who reported a diagnosis of diabetes, 184 (80%) were seen at the latest follow-up, when 172 (75%) gave permission to contact their GP. A validation questionnaire was developed and sent to the GP of all consenting participants with a self-reported diabetes diagnosis. The questionnaire consisted of questions on diabetes status and type, date of diagnosis, how the diagnosis was established and which type of treatment patients were currently receiving (diet, oral hypoglycaemic agents, insulin or other). The validity of self-reported diabetes was assessed by calculating the percentage of self-reported diabetes cases that were confirmed to have diabetes by their GP, i.e. the positive predictive value (PPV) with GP confirmation as the gold standard (PPV = *b*/*a* × 100, where *a* = number self-reported and *b* = those confirmed by GP). The difference between self-reported and GP-confirmed age at diagnosis was analysed with a Bland–Altman plot [16]; the mean difference, 95% CI and limits of agreements were calculated.

## 3 Results

Completed questionnaires were obtained from 157 GPs (91.2%). Of these, 149 self-reported diagnoses were confirmed by the GP (PPV = 94.9%). Results were very similar when the analyses were performed using only responses to a direct question on diabetes diagnosis (PPV = 95.4%). Of the GP-confirmed cases 143 (95.9%) were type 2 diabetes and six (4%) were type 1 diabetes. Of the eight cases that were not confirmed two had pre-diabetes (GP-reported impaired fasting glycaemia or glucose intolerance). Of the remaining six, four reported having been diagnosed at age 26, 49, 61 and 62 years and two said they were diagnosed between 53 and 63 years. Of these six, three said they were prescribed a diabetic diet, but no tablets, from their doctors, and one had FPG > 7 mmol/L at the NSHD 2006–11 data collection round. Information on the test used to diagnose diabetes was available for 121 participants. Fasting plasma glucose (FPG) (*n* = 68, 56.2%) and oral glucose tolerance (OGT) (*n* = 15, 12.4%) were the most common tests. FPG was also used in combination with OGT by 10 (8.26%) GPs and in combination with other tests (usually HbA1c) by 14 (11.5%) GPs. For the remaining 14 study members (11.5%) diagnosis was made because of high random blood glucose (range 7.1–48.6 mmol/L), alone or together with high HbA1c (>10% or 86 mmol/mol) and symptoms.

**Fig. 1:**
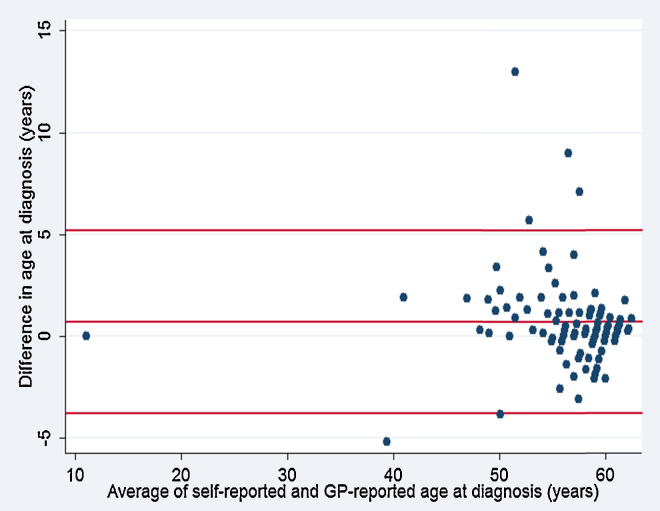
Differences in years between self-reported and GP-confirmed age at diagnosis plotted against the average difference. Horizontal lines denote the mean difference (0.6 years), and the upper (5.1 years) and lower (−3.7 years) limits of agreement (mean difference ± 1.96 SD of the differences).

The date of diagnosis was reported by 148 GPs. The mean age at diagnosis was 55.5 years (±SD 7.3). Information on self-reported age at diagnosis was available for 102 study members. Of these, 37 (36.2%) reported the same age in years at diagnosis as their GP. Fig. 1 plots the differences between self-reported and GP-reported age at diagnosis against the average difference. The average difference was 0.6 years (95% CI 0.2–1.1). The 95% limits of agreements were 5.1/−3.7 years. Information on treatment was reported by 148 GPs. The combination of diet and oral hypoglycaemic agents was the most common treatment prescribed (37.1%) followed by oral hypoglycaemic agents alone (31%) and diet alone (15.5%). Twenty-four (16.2%) study members were treated with insulin.

## 4 Discussion

In the present study the proportion of self-reported diabetes in a representative population-based UK sample that was confirmed by GPs was very high (PPV = 94.9%). A few studies have reported good agreement between self-reported diabetes diagnosis and medical records, with kappa (*κ*) values of agreement and PPV ranging between 0.71 and 0.94 [6,9,10,14]. However, none of these have been conducted among the general British population. The result of the present study was similar to previous non-British diabetes validation studies that used family doctors as the gold standard [1,9,10,14]. It has been suggested that the high agreement of self-reported diabetes might be partly due to the well-defined diagnostic criteria of this disease and to the fact that it often requires treatment once diagnosed [9,10]. This study found that the self-reported age at diagnosis was between 0.2 and 1.1 years earlier than the age reported by the GP. This result is similar to previous studies, which indicated that patients tend to overestimate the duration of their condition [1,2].

Two strengths of this study were the use of a representative sample of the general British population and the excellent GP response rate (91.2%). The high response rate could be attributed to the financial incentive offered to the GP for returning a complete questionnaire and the repeated attempts, either by phone or mail, to contact the GPs and the study members. However, this study also had two weaknesses. Only the GPs of study members who said they had diabetes were contacted; thus the number of study members who did not self-reported diabetes, but who, according to GP records, had been diagnosed was unknown in this study. Another weakness was the fact that all the participants were ethnically white, which means the results cannot be generalised to ethnicities with higher risk of diabetes.

## 5 Summary

In conclusion, this study suggests that self-reported diabetes in the UK population, as represented by responses to a direct question among the British population, is generally confirmed by GP records. Although these questionnaires did not provide information on undiagnosed diabetes, they may be used as an inexpensive and convenient method of diabetes assessment in epidemiological studies.

## Conflict of interest

The authors state that they have no conflict of interest.

## Figures and Tables

**Table 1:** Participants available for validation and GP response rate.

	No.	%
Total self-reported diabetes 1977–2008	230	
Died	19	
Withdrew	9	
Lost to follow up	15	
Emigrated	2	
Seen at the latest follow-up	184	
Refused consent to contact their GP	7	
Died after follow-up	5	
Available for validation study	172	74.7
1st questionnaire sent to GPs	172	
GPs telephoned	27	
Study members telephoned	11	
Questionnaire resent to GPs	24	
Questionnaire sent to new GPs	11	
Questionnaires returned (GP response rate)	**157**	**91.2**

GP = general practitioner.
